# Tris[tris­(ethane-1,2-diamine)cobalt(II)] bis­[octa­cyanidomolybdate(V)] dihydrate

**DOI:** 10.1107/S1600536808024951

**Published:** 2008-08-13

**Authors:** Chao-Xia Chu, Hu Zhou, Lang Liu, Ai-Hua Yuan

**Affiliations:** a School of Materials Science and Engineering, Jiangsu University of Science and Technology, Zhenjiang 212003, People’s Republic of China; b Institute of Applied Chemistry, Xinjiang University, Urumqi 830046, Xinjiang, People’s Republic of China

## Abstract

In the title compound, [Co^II^(C_2_H_8_N_2_)_3_]_3_[Mo^V^(CN)_8_]_2_·2H_2_O, N—H⋯N and N—H⋯O hydrogen-bonding inter­actions give rise to a three-dimensional network. In the crystal structure, each Mo polyhedron has a square-anti­prismatic shape, while the Co complexes show distorted octa­hedral geometry with an occupancy of 50%. One of the Co atoms resides on a crystallographic inversion centre.

## Related literature

For information on octa­cyanido­metalate-based compounds, see: Bok *et al.* (1975 ▶); Lim *et al.* (2006 ▶) and literature cited therein; Przychodzeń *et al.* (2006 ▶) and literature cited therein; Sieklucka *et al.* (2002 ▶); Willemin *et al.* (2003 ▶); Withers *et al.* (2006 ▶). For related literature, see: Aschwanden *et al.* (1993 ▶); Müller *et al.* (2006 ▶).
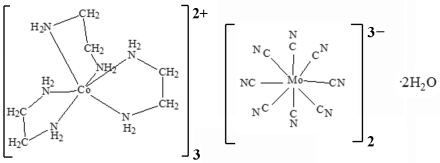


## Experimental

### 

#### Crystal data

[Co(C_2_H_8_N_2_)_3_]_3_[Mo(CN)_8_]_2_·2H_2_O
*M* *_r_* = 1361.96
Monoclinic, 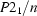

*a* = 9.2113 (3) Å
*b* = 30.5439 (8) Å
*c* = 11.4022 (3) Å
β = 94.138 (1)°
*V* = 3199.63 (16) Å^3^
*Z* = 2
Mo *K*α radiation
μ = 1.20 mm^−1^
*T* = 153 (2) K
0.25 × 0.23 × 0.18 mm

#### Data collection

Rigaku R-AXIS Spider diffractometer
Absorption correction: multi-scan (*ABSCOR*; Higashi, 1995 ▶) *T*_min_ = 0.753, *T*_max_ = 0.813
27139 measured reflections
6276 independent reflections
4952 reflections with *I* > 2σ(*I*)
*R*_int_ = 0.054

#### Refinement

*R*[*F* ^2^ > 2σ(*F* ^2^)] = 0.047
*wR*(*F* ^2^) = 0.101
*S* = 1.06
6276 reflections
415 parameters
56 restraints
H-atom parameters constrained
Δρ_max_ = 0.42 e Å^−3^
Δρ_min_ = −0.55 e Å^−3^

### 

Data collection: *RAPID-AUTO* (Rigaku, 2004 ▶); cell refinement: *RAPID-AUTO*; data reduction: *RAPID-AUTO*; program(s) used to solve structure: *SHELXS97* (Sheldrick, 2008 ▶); program(s) used to refine structure: *SHELXL97* (Sheldrick, 2008 ▶); molecular graphics: *SHELXTL* (Sheldrick, 2008 ▶); software used to prepare material for publication: *SHELXL97* and *PLATON* (Spek, 2003 ▶).

## Supplementary Material

Crystal structure: contains datablocks global, I. DOI: 10.1107/S1600536808024951/si2095sup1.cif

Structure factors: contains datablocks I. DOI: 10.1107/S1600536808024951/si2095Isup2.hkl

Additional supplementary materials:  crystallographic information; 3D view; checkCIF report

## Figures and Tables

**Table 1:** Hydrogen-bond geometry (Å, °)

*D*—H⋯*A*	*D*—H	H⋯*A*	*D*⋯*A*	*D*—H⋯*A*
N9—H9*C*⋯N7^i^	0.92	2.24	3.064 (4)	148
N11—H11*C*⋯N7^i^	0.92	2.09	2.985 (4)	165
N12—H12*C*⋯N6^ii^	0.92	2.10	2.955 (4)	154
N13—H13*C*⋯N8^ii^	0.92	2.55	3.138 (4)	122
N9—H9*D*⋯N2	0.92	2.32	3.117 (4)	145
N14—H14*D*⋯N1	0.92	2.28	3.130 (5)	154
N10—H10*C*⋯O3^iii^	0.92	1.96	2.856 (10)	164
N10—H10*D*⋯N5^iii^	0.92	2.49	3.125 (5)	126
N11—H11*D*⋯N6^iii^	0.92	2.58	3.163 (4)	122
N13—H13*D*⋯N5^iii^	0.92	2.11	3.012 (4)	167
N16—H16*C*⋯N4^iv^	0.92	2.52	3.19 (2)	130
N17—H17*A*⋯N2^v^	0.97	2.49	3.29 (2)	140
N20—H20*C*⋯N2^iv^	0.85	2.44	3.02 (2)	127
O2—H2*A*⋯N8^vi^	0.85 (14)	2.41 (14)	3.207 (9)	156 (12)
O2—H2*B*⋯N2^vi^	0.85 (15)	2.50 (15)	3.245 (9)	147 (12)

Symmetry codes: (i) 
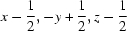
; (ii) 
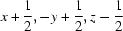
; (iii) 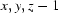; (iv) 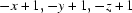; (v) 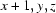; (vi) 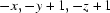.

**Fig. 1.**
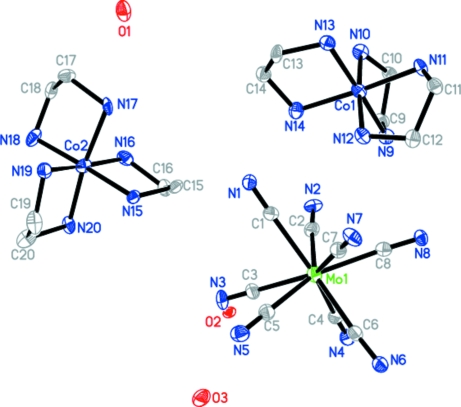
ORTEP view of the title compound showing 30% probability probability displacement ellipsoids and atom labelling scheme. Hydrogen atoms have been omitted for clarity. The occupancies of the water oxygen atoms are 0.4 (O1), 0.3 (O2), 0.3 (O3).

**Fig. 2.**
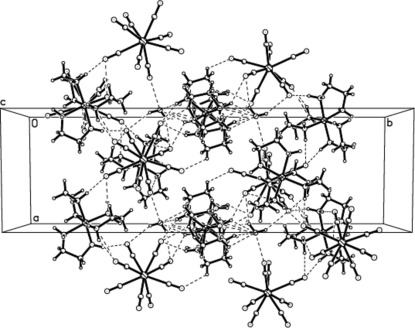
Packing diagram of the title compound presenting a three-dimensional hydrogen bonding network.

## supplementary crystallographic information

### Comment

Recently, octacyanometallates [*M*(CN)_8_]^3-/4-^ (*M* = Mo and W) appear as new versatile building blocks and have been investigated extensively (Przychodzeń *et al.*, 2006; Sieklucka *et al.*, 2002). These species might show various geometrical structures (*e.g.*, square antiprism, dodecahedron, bicapped trigonal prism) depending on the external environments. However, only a few examples of cobalt-octacyanometalate coordination networks have been reported until now (Willemin *et al.*, 2003; Przychodzeń *et al.*, 2006 and literature cited therein). In the title bimetallic compound, [Co^II^(en)_3_]_3_[Mo^V^(CN)_8_]_2_.2H_2_O, (Fig. 1), the Mo^V^ atom is coordinated by eight CN groups with Mo—C distances ranging from 2.128 (4) to 2.179 (4) Å in a distorted square antiprism. The Mo^V^—C bond distances are comparable to previously reported values (Withers *et al.*, 2006; Lim *et al.*, 2006 and literature cited therein). The geometry around atom Co1 is a distorted octahedron with (D*_3_* symmetry) with an average Co1—N bond distance 1.972 Å and the N—Co1—N bond angles ranging from 84.34 (12)° to 176.73 (12)°. Among the two independent Co(en)_3_ complexes the cobalt atom Co2 resides on a crystallographic inversion centre, resulting in inversion related 50:50 disorder of the chiral Λ and Δ forms. Disorder refinement (Müller *et al.* 2006) was necessary to present a suitable chiral model of the complex. (Fig. 1).

Several classic intermolecular N—H···N hydrogen bonds (Fig. 2) between the non-disordered Co(en)_3_ and the Mo(CN)_8_ complexes form a complicated three-dimensional network in the structure. The disordered Co2 unit and the disordered water molecules are not considered for further (possible) hydrogen bonding contacts. An interesting example structure with two crystallographically independent Co(en)_3_ complexes, different vanadates and six water molecules shows an impressive number of 23 N—H···O and 13 O—H···O hydrogen bonds in the chiral space group P1 (Aschwanden *et al.* 1993). It may be thinkable that the title structure belongs also to a non-centrosymmetric space group (P 21), but in that case with a dominating part of centrosymmetry in data.

### Experimental

For the preparation of the title compound, all of the following procedures were carried out in the dark to avoid decomposition of (Bu_3_NH)_3_[Mo(CN)_8_].4H_2_O (Bok *et al.*, 1975). Yellow block crystals suitable for X-ray single-crystal structure determination were grown at room temperature by slow diffusion of an aqueous solution of CoCl_2_.6H_2_O (0.3 mmol) and ethane-1,2-amine (en, 0.9 mmol) and an aqueous solution of (Bu_3_NH)_3_[Mo(CN)_8_].4H_2_O (0.2 mmol) for four weeks. The resulting crystals were collected, washed with H_2_O and dried in air.

### Refinement

All non-H atoms were refined anisotropically. The H atoms on nitrogen atoms were located from the difference Fourier maps, and the H atoms of water molecules were placed in calculated positions. The H atoms of the Co complexes were placed in calculated positions with C—H and N—H distances 0.99 Å and 0.92 Å, respectively, with *U*_iso_(H) = 1.2*U*_eq_(C,*N*). The calculation of the H atoms for the disordered Co2 complex was possible with many FREE instructions using *SHELXL97* (Sheldrick, 2008). For atoms N17 and N20 the four H-atom coordinates from the difference map were fixed with AFIX 3 instructions. These N—H distances range between 0.767 and 0.968 Å. The model refinement of the Co2(en)_3_ complex was controlled with the programme *PLATON* (Spek, 2003), (LATT -1 used for model building at the start), symmetry transformation (2 - *x*, 1 - *y*, 1 - *z*) for some atom coordinates applied, introducing a split position for Co2 to get the complete Λ and Δ forms of the inversion related chiral complexes separated, using the information of disorder refinement from the *SHELXL* guide book (Müller *et al.*, 2006).

### Crystal data

**Table:**

[Co(C_2_H_8_N_2_)_3_]_3_[Mo(CN)_8_]_2_·2H_2_O	*F*_000_ = 1398
*M**_r_* = 1361.96	*D*_x_ = 1.414 Mg m^−^^3^
Monoclinic, *P*2_1_/*n*	Mo *K*α radiation λ = 0.71073 Å
Hall symbol: -P 2yn	Cell parameters from 7571 reflections
*a* = 9.2113 (3) Å	θ = 2.1–26.4º
*b* = 30.5439 (8) Å	µ = 1.20 mm^−^^1^
*c* = 11.4022 (3) Å	*T* = 153 (2) K
β = 94.1380 (10)º	Block, yellow
*V* = 3199.63 (16) Å^3^	0.25 × 0.23 × 0.18 mm
*Z* = 2	

### Data collection

**Table:**

Bruker SMART CCD diffractometer	6276 independent reflections
Radiation source: sealed tube	4952 reflections with *I* > 2σ(*I*)
Monochromator: graphite	*R*_int_ = 0.054
*T* = 153(2) K	θ_max_ = 26.0º
φ and ω scans	θ_min_ = 3.0º
Absorption correction: multi-scan(SADABS; Bruker, 2000)	*h* = −11→11
*T*_min_ = 0.754, *T*_max_ = 0.813	*k* = −37→37
27139 measured reflections	*l* = −14→14

### Refinement

**Table:**

Refinement on *F*^2^	Secondary atom site location: difference Fourier map
Least-squares matrix: full	Hydrogen site location: inferred from neighbouring sites
*R*[*F*^2^ > 2σ(*F*^2^)] = 0.047	H-atom parameters constrained
*wR*(*F*^2^) = 0.101	*w* = 1/[σ^2^(*F*_o_^2^) + (0.0504*P*)^2^] where *P* = (*F*_o_^2^ + 2*F*_c_^2^)/3
*S* = 1.06	(Δ/σ)_max_ < 0.001
6276 reflections	Δρ_max_ = 0.42 e Å^−^^3^
415 parameters	Δρ_min_ = −0.55 e Å^−^^3^
56 restraints	Extinction correction: none
Primary atom site location: structure-invariant direct methods	

### Special details

**Table:**

Geometry. All e.s.d.'s (except the e.s.d. in the dihedral angle between two l.s. planes) are estimated using the full covariance matrix. The cell e.s.d.'s are taken into account individually in the estimation of e.s.d.'s in distances, angles and torsion angles; correlations between e.s.d.'s in cell parameters are only used when they are defined by crystal symmetry. An approximate (isotropic) treatment of cell e.s.d.'s is used for estimating e.s.d.'s involving l.s. planes.
Refinement. Refinement of *F*^2^ against ALL reflections. The weighted *R*-factor *wR* and goodness of fit *S* are based on *F*^2^, conventional *R*-factors *R* are based on *F*, with *F* set to zero for negative *F*^2^. The threshold expression of *F*^2^ > σ(*F*^2^) is used only for calculating *R*-factors(gt) *etc*. and is not relevant to the choice of reflections for refinement. *R*-factors based on *F*^2^ are statistically about twice as large as those based on *F*, and *R*- factors based on ALL data will be even larger.

### Fractional atomic coordinates and isotropic or equivalent isotropic displacement parameters (Å^2^)

**Table:**

	*x*	*y*	*z*	*U*_iso_*/*U*_eq_	Occ. (<1)
Mo1	0.40449 (4)	0.336480 (9)	0.62354 (3)	0.03307 (10)	
C1	0.5956 (4)	0.34579 (12)	0.5242 (3)	0.0339 (7)	
N1	0.6910 (3)	0.34961 (9)	0.4732 (3)	0.0329 (6)	
C2	0.3472 (4)	0.37899 (11)	0.4797 (3)	0.0354 (8)	
N2	0.3071 (4)	0.40123 (10)	0.3965 (3)	0.0374 (7)	
C3	0.4699 (4)	0.40112 (12)	0.6844 (3)	0.0380 (8)	
N3	0.5017 (4)	0.43479 (9)	0.7151 (3)	0.0406 (8)	
C4	0.2090 (4)	0.36648 (11)	0.6775 (3)	0.0313 (7)	
N4	0.1008 (4)	0.38188 (9)	0.7010 (3)	0.0385 (7)	
C5	0.5642 (4)	0.33094 (11)	0.7700 (3)	0.0335 (8)	
N5	0.6477 (4)	0.32846 (11)	0.8481 (3)	0.0399 (7)	
C6	0.3104 (4)	0.29450 (11)	0.7497 (3)	0.0328 (7)	
N6	0.2601 (3)	0.26949 (10)	0.8135 (3)	0.0341 (6)	
C7	0.4994 (4)	0.27234 (11)	0.5986 (3)	0.0327 (7)	
N7	0.5468 (3)	0.23832 (10)	0.5864 (3)	0.0357 (7)	
N8	0.1493 (3)	0.28720 (9)	0.4532 (2)	0.0307 (6)	
C8	0.2437 (4)	0.30333 (11)	0.5112 (3)	0.0327 (7)	
Co1	0.44625 (5)	0.313270 (15)	0.15051 (4)	0.03194 (13)	
N9	0.2527 (3)	0.33083 (9)	0.2003 (3)	0.0327 (6)	
H9C	0.1911	0.3071	0.1969	0.039*	
H9D	0.2616	0.3408	0.2766	0.039*	
C9	0.1923 (4)	0.36582 (11)	0.1216 (3)	0.0349 (8)	
H9A	0.2359	0.3943	0.1464	0.042*	
H9B	0.0854	0.3678	0.1250	0.042*	
C10	0.2286 (4)	0.35532 (12)	0.0022 (3)	0.0323 (7)	
H10A	0.2033	0.3802	−0.0510	0.039*	
H10B	0.1731	0.3293	−0.0271	0.039*	
N10	0.3897 (3)	0.34622 (9)	0.0052 (3)	0.0350 (7)	
H10C	0.4406	0.3721	0.0044	0.042*	
H10D	0.4105	0.3301	−0.0596	0.042*	
N11	0.3701 (3)	0.25818 (9)	0.0810 (3)	0.0345 (7)	
H11C	0.2706	0.2601	0.0678	0.041*	
H11D	0.4087	0.2537	0.0097	0.041*	
C11	0.4077 (4)	0.21994 (11)	0.1607 (3)	0.0327 (7)	
H11A	0.5070	0.2091	0.1497	0.039*	
H11B	0.3375	0.1957	0.1457	0.039*	
C12	0.3977 (4)	0.23912 (11)	0.2871 (3)	0.0324 (7)	
H12A	0.2948	0.2453	0.3011	0.039*	
H12B	0.4361	0.2177	0.3466	0.039*	
N12	0.4821 (3)	0.27916 (9)	0.2972 (2)	0.0304 (6)	
H12C	0.5796	0.2726	0.3091	0.036*	
H12D	0.4554	0.2953	0.3603	0.036*	
N13	0.6390 (3)	0.29952 (9)	0.1000 (2)	0.0300 (6)	
H13C	0.6599	0.2706	0.1156	0.036*	
H13D	0.6395	0.3037	0.0201	0.036*	
C13	0.7505 (4)	0.32704 (10)	0.1603 (3)	0.0333 (8)	
H13A	0.8376	0.3286	0.1145	0.040*	
H13B	0.7797	0.3150	0.2391	0.040*	
C14	0.6848 (4)	0.37129 (11)	0.1710 (3)	0.0351 (8)	
H14A	0.7492	0.3900	0.2230	0.042*	
H14B	0.6716	0.3854	0.0928	0.042*	
N14	0.5431 (3)	0.36574 (9)	0.2211 (3)	0.0345 (7)	
H14C	0.4861	0.3901	0.2053	0.041*	
H14D	0.5563	0.3625	0.3014	0.041*	
Co2	0.9953 (18)	0.5039 (4)	0.4938 (14)	0.0293 (10)	0.50
N15	0.8779 (19)	0.4547 (8)	0.5513 (12)	0.029 (3)	0.50
H15A	0.9260	0.4286	0.5442	0.035*	0.50
H15B	0.8607	0.4588	0.6290	0.035*	0.50
C15	0.7350 (7)	0.4548 (2)	0.4745 (6)	0.0330 (15)	0.50
H15C	0.7487	0.4396	0.3994	0.040*	0.50
H15D	0.6595	0.4389	0.5151	0.040*	0.50
C16	0.6874 (8)	0.5000 (2)	0.4509 (7)	0.0376 (16)	0.50
H16A	0.6584	0.5139	0.5241	0.045*	0.50
H16B	0.6027	0.5003	0.3924	0.045*	0.50
N16	0.8157 (18)	0.5252 (7)	0.4031 (15)	0.037 (3)	0.50
H16C	0.8045	0.5548	0.4136	0.044*	0.50
H16D	0.8208	0.5197	0.3242	0.044*	0.50
N17	1.0281 (19)	0.4680 (8)	0.3505 (16)	0.031 (3)	0.50
H17A	1.0698	0.4407	0.3802	0.037*	0.50
H17B	0.9572	0.4652	0.3117	0.037*	0.50
C17	1.1534 (9)	0.4942 (2)	0.3021 (6)	0.0366 (16)	0.50
H17C	1.2463	0.4872	0.3471	0.044*	0.50
H17D	1.1628	0.4866	0.2186	0.044*	0.50
C18	1.1218 (8)	0.5402 (2)	0.3126 (6)	0.0364 (16)	0.50
H18A	1.0262	0.5469	0.2711	0.044*	0.50
H18B	1.1969	0.5577	0.2759	0.044*	0.50
N18	1.119 (2)	0.5524 (8)	0.4429 (15)	0.046 (6)	0.50
H18C	1.0773	0.5793	0.4531	0.055*	0.50
H18D	1.2108	0.5518	0.4810	0.055*	0.50
N19	1.1687 (17)	0.4768 (7)	0.5739 (13)	0.033 (4)	0.50
H19A	1.1711	0.4470	0.5628	0.040*	0.50
H19B	1.2538	0.4892	0.5523	0.040*	0.50
C19	1.1358 (9)	0.4890 (2)	0.6990 (8)	0.045 (2)	0.50
H19C	1.0650	0.4676	0.7262	0.054*	0.50
H19D	1.2267	0.4858	0.7502	0.054*	0.50
C20	1.0790 (9)	0.5325 (2)	0.7165 (8)	0.047 (2)	0.50
H20A	1.1496	0.5551	0.6956	0.057*	0.50
H20B	1.0564	0.5368	0.7993	0.057*	0.50
N20	0.9413 (19)	0.5345 (9)	0.6346 (16)	0.032 (4)	0.50
H20C	0.9271	0.5615	0.6229	0.038*	0.50
H20D	0.8796	0.5215	0.6781	0.038*	0.50
O1	0.9811 (9)	0.5154 (2)	0.0352 (6)	0.055 (2)	0.40
H1A	1.0629	0.5055	0.0173	0.066*	0.40
H1B	0.9246	0.5179	−0.0268	0.066*	0.40
O2	0.0345 (9)	0.6253 (3)	0.6065 (6)	0.0315 (17)	0.30
H2A	0.0124	0.6518	0.5917	0.038*	0.30
H2B	−0.0393	0.6092	0.5896	0.038*	0.30
O3	0.4984 (11)	0.4328 (3)	0.9766 (8)	0.048 (2)	0.30
H3D	0.4504	0.4525	0.9384	0.058*	0.30
H3C	0.5859	0.4412	0.9908	0.058*	0.30

### Atomic displacement parameters (Å^2^)

**Table:**

	*U*^11^	*U*^22^	*U*^33^	*U*^12^	*U*^13^	*U*^23^
Mo1	0.03407 (18)	0.02993 (16)	0.03468 (17)	−0.00118 (12)	−0.00121 (12)	0.00009 (12)
C1	0.0324 (19)	0.0422 (19)	0.0274 (17)	−0.0018 (15)	0.0042 (14)	−0.0041 (15)
N1	0.0323 (16)	0.0348 (15)	0.0314 (15)	−0.0073 (12)	0.0012 (13)	0.0006 (12)
C2	0.042 (2)	0.0281 (16)	0.0366 (19)	−0.0046 (14)	0.0066 (16)	0.0034 (15)
N2	0.0466 (19)	0.0355 (15)	0.0317 (16)	0.0061 (13)	0.0142 (14)	0.0055 (13)
C3	0.043 (2)	0.0377 (19)	0.0322 (19)	−0.0108 (16)	−0.0009 (16)	−0.0008 (15)
N3	0.0393 (18)	0.0302 (16)	0.0505 (19)	−0.0123 (13)	−0.0100 (14)	−0.0040 (14)
C4	0.037 (2)	0.0283 (16)	0.0288 (17)	0.0002 (14)	0.0031 (14)	0.0001 (14)
N4	0.0437 (19)	0.0296 (14)	0.0425 (18)	0.0004 (13)	0.0057 (14)	−0.0006 (13)
C5	0.0323 (19)	0.0338 (17)	0.0343 (19)	0.0010 (14)	0.0029 (15)	−0.0022 (15)
N5	0.0345 (17)	0.0517 (18)	0.0333 (16)	0.0051 (14)	0.0015 (14)	0.0048 (14)
C6	0.0351 (19)	0.0343 (17)	0.0282 (17)	−0.0028 (15)	−0.0027 (14)	−0.0049 (15)
N6	0.0363 (17)	0.0363 (15)	0.0290 (15)	−0.0033 (13)	−0.0023 (12)	−0.0003 (13)
C7	0.0355 (19)	0.0335 (18)	0.0284 (17)	−0.0013 (14)	−0.0022 (14)	0.0047 (14)
N7	0.0304 (16)	0.0426 (17)	0.0325 (16)	0.0137 (13)	−0.0092 (12)	−0.0091 (13)
N8	0.0246 (14)	0.0328 (14)	0.0336 (15)	−0.0002 (11)	−0.0044 (12)	−0.0045 (12)
C8	0.0338 (19)	0.0347 (17)	0.0297 (17)	0.0036 (14)	0.0041 (14)	−0.0001 (14)
Co1	0.0350 (3)	0.0350 (2)	0.0252 (2)	−0.00011 (19)	−0.00237 (18)	−0.00180 (19)
N9	0.0306 (16)	0.0331 (14)	0.0340 (15)	0.0076 (12)	0.0004 (12)	0.0029 (12)
C9	0.0355 (19)	0.0327 (17)	0.0363 (19)	0.0052 (15)	0.0015 (15)	−0.0089 (15)
C10	0.0300 (18)	0.0378 (18)	0.0282 (18)	0.0065 (14)	−0.0040 (13)	−0.0036 (14)
N10	0.0396 (17)	0.0286 (14)	0.0366 (16)	0.0045 (12)	0.0016 (13)	0.0021 (12)
N11	0.0402 (18)	0.0293 (14)	0.0327 (15)	−0.0047 (12)	−0.0059 (12)	−0.0090 (12)
C11	0.0361 (19)	0.0322 (16)	0.0288 (17)	−0.0001 (14)	−0.0046 (14)	0.0002 (14)
C12	0.0277 (18)	0.0329 (17)	0.0354 (18)	0.0018 (13)	−0.0069 (14)	0.0068 (14)
N12	0.0320 (15)	0.0283 (13)	0.0295 (14)	0.0054 (11)	−0.0066 (11)	0.0006 (12)
N13	0.0299 (15)	0.0325 (14)	0.0272 (14)	0.0038 (11)	−0.0016 (11)	0.0003 (12)
C13	0.0350 (19)	0.0296 (16)	0.0343 (18)	−0.0158 (14)	−0.0041 (14)	0.0134 (14)
C14	0.037 (2)	0.0371 (18)	0.0306 (18)	−0.0040 (15)	−0.0026 (15)	0.0032 (15)
N14	0.0287 (16)	0.0349 (15)	0.0392 (17)	0.0006 (12)	−0.0018 (13)	0.0020 (13)
Co2	0.0417 (17)	0.014 (3)	0.033 (3)	−0.001 (2)	0.0104 (15)	0.007 (2)
N15	0.033 (7)	0.021 (7)	0.034 (6)	−0.008 (4)	0.013 (5)	−0.007 (4)
C15	0.033 (4)	0.027 (3)	0.041 (4)	−0.007 (3)	0.019 (3)	−0.008 (3)
C16	0.033 (4)	0.036 (4)	0.043 (4)	0.001 (3)	−0.005 (3)	−0.003 (3)
N16	0.051 (7)	0.032 (5)	0.025 (6)	0.000 (4)	−0.004 (5)	0.004 (5)
N17	0.015 (7)	0.033 (6)	0.042 (6)	0.002 (5)	−0.011 (5)	−0.014 (5)
C17	0.047 (4)	0.033 (4)	0.031 (4)	−0.001 (3)	0.012 (3)	0.002 (3)
C18	0.027 (4)	0.039 (4)	0.042 (4)	−0.010 (3)	−0.002 (3)	0.011 (3)
N18	0.039 (8)	0.025 (7)	0.078 (11)	−0.012 (4)	0.029 (7)	−0.019 (6)
N19	0.037 (6)	0.035 (6)	0.028 (7)	−0.008 (4)	−0.003 (4)	0.010 (4)
C19	0.040 (4)	0.025 (3)	0.067 (6)	0.002 (3)	−0.014 (4)	0.006 (4)
C20	0.039 (5)	0.042 (4)	0.063 (5)	0.015 (3)	0.014 (4)	0.014 (4)
N20	0.018 (7)	0.033 (4)	0.042 (6)	0.010 (5)	−0.010 (5)	−0.004 (4)
O1	0.058 (5)	0.056 (4)	0.048 (4)	−0.020 (4)	−0.016 (4)	0.013 (3)
O2	0.033 (4)	0.037 (4)	0.024 (4)	−0.004 (3)	0.003 (3)	0.013 (3)
O3	0.055 (6)	0.041 (5)	0.049 (5)	−0.010 (4)	0.011 (4)	0.009 (4)

### Geometric parameters (Å, °)

**Table:**

Mo1—C2	2.128 (4)	C14—H14A	0.9900
Mo1—C8	2.142 (4)	C14—H14B	0.9900
Mo1—C4	2.150 (4)	N14—H14C	0.9200
Mo1—C5	2.151 (4)	N14—H14D	0.9200
Mo1—C6	2.155 (4)	Co2—Co2^i^	0.290 (16)
Mo1—C3	2.164 (4)	Co2—N20	1.95 (2)
Mo1—C7	2.172 (3)	Co2—N19	1.966 (12)
Mo1—C1	2.179 (4)	Co2—N18	1.98 (2)
C1—N1	1.095 (5)	Co2—N15	1.990 (18)
C2—N2	1.204 (5)	Co2—N16	1.994 (15)
C3—N3	1.119 (4)	Co2—N17	2.010 (19)
C4—N4	1.151 (5)	N15—C15	1.528 (17)
C5—N5	1.137 (5)	N15—H15A	0.9200
C6—N6	1.173 (5)	N15—H15B	0.9200
C7—N7	1.140 (4)	C15—C16	1.468 (8)
N8—C8	1.163 (4)	C15—H15C	0.9900
Co1—N13	1.952 (3)	C15—H15D	0.9900
Co1—N11	1.968 (3)	C16—N16	1.542 (17)
Co1—N10	1.976 (3)	C16—H16A	0.9900
Co1—N14	1.977 (3)	C16—H16B	0.9900
Co1—N12	1.978 (3)	N16—H16C	0.9200
Co1—N9	1.983 (3)	N16—H16D	0.9200
N9—C9	1.478 (4)	N17—C17	1.540 (18)
N9—H9C	0.9200	N17—H17A	0.968
N9—H9D	0.9200	N17—H17B	0.767
C9—C10	1.460 (5)	C17—C18	1.441 (9)
C9—H9A	0.9900	C17—H17C	0.9900
C9—H9B	0.9900	C17—H17D	0.9900
C10—N10	1.507 (4)	C18—N18	1.534 (16)
C10—H10A	0.9900	C18—H18A	0.9900
C10—H10B	0.9900	C18—H18B	0.9900
N10—H10C	0.9200	N18—H18C	0.9200
N10—H10D	0.9200	N18—H18D	0.9200
N11—C11	1.505 (4)	N19—C19	1.526 (12)
N11—H11C	0.9200	N19—H19A	0.9200
N11—H11D	0.9200	N19—H19B	0.9200
C11—C12	1.565 (5)	C19—C20	1.445 (8)
C11—H11A	0.9900	C19—H19C	0.9900
C11—H11B	0.9900	C19—H19D	0.9900
C12—N12	1.450 (4)	C20—N20	1.522 (11)
C12—H12A	0.9900	C20—H20A	0.9900
C12—H12B	0.9900	C20—H20B	0.9900
N12—H12C	0.9200	N20—H20C	0.845
N12—H12D	0.9200	N20—H20D	0.876
N13—C13	1.460 (4)	O1—O1^ii^	1.299 (16)
N13—H13C	0.9200	O1—H1A	0.85
N13—H13D	0.9200	O1—H1B	0.85
C13—C14	1.489 (5)	O2—H2A	0.85
C13—H13A	0.9900	O2—H2B	0.85
C13—H13B	0.9900	O3—H3D	0.85
C14—N14	1.472 (5)	O3—H3C	0.85
			
C2—Mo1—C8	72.86 (13)	C14—C13—H13A	110.4
C2—Mo1—C4	78.12 (14)	N13—C13—H13B	110.4
C8—Mo1—C4	79.35 (13)	C14—C13—H13B	110.4
C2—Mo1—C5	139.82 (14)	H13A—C13—H13B	108.6
C8—Mo1—C5	146.29 (13)	N14—C14—C13	107.7 (3)
C4—Mo1—C5	110.56 (13)	N14—C14—H14A	110.2
C2—Mo1—C6	141.99 (14)	C13—C14—H14A	110.2
C8—Mo1—C6	79.84 (13)	N14—C14—H14B	110.2
C4—Mo1—C6	71.15 (13)	C13—C14—H14B	110.2
C5—Mo1—C6	73.77 (13)	H14A—C14—H14B	108.5
C2—Mo1—C3	74.81 (13)	C14—N14—Co1	108.8 (2)
C8—Mo1—C3	141.97 (14)	C14—N14—H14C	109.9
C4—Mo1—C3	74.87 (14)	Co1—N14—H14C	109.9
C5—Mo1—C3	70.36 (14)	C14—N14—H14D	109.9
C6—Mo1—C3	116.44 (13)	Co1—N14—H14D	109.9
C2—Mo1—C7	121.95 (13)	H14C—N14—H14D	108.3
C8—Mo1—C7	76.28 (13)	N20—Co2—N19	93.8 (10)
C4—Mo1—C7	140.82 (13)	N20—Co2—N18	94.0 (9)
C5—Mo1—C7	76.81 (13)	N19—Co2—N18	89.1 (13)
C6—Mo1—C7	74.67 (13)	N20—Co2—N15	85.0 (12)
C3—Mo1—C7	139.42 (14)	N19—Co2—N15	88.3 (8)
C2—Mo1—C1	71.62 (14)	N18—Co2—N15	177.2 (13)
C8—Mo1—C1	107.49 (13)	N20—Co2—N16	91.1 (8)
C4—Mo1—C1	144.87 (13)	N19—Co2—N16	173.6 (15)
C5—Mo1—C1	83.18 (13)	N18—Co2—N16	94.5 (8)
C6—Mo1—C1	143.45 (14)	N15—Co2—N16	88.1 (12)
C3—Mo1—C1	80.26 (14)	N20—Co2—N17	172.8 (15)
C7—Mo1—C1	72.66 (13)	N19—Co2—N17	89.0 (8)
N1—C1—Mo1	178.5 (3)	N18—Co2—N17	92.6 (11)
N2—C2—Mo1	175.7 (3)	N15—Co2—N17	88.4 (8)
N3—C3—Mo1	178.9 (4)	N16—Co2—N17	85.6 (11)
N4—C4—Mo1	176.5 (3)	C15—N15—Co2	106.0 (12)
N5—C5—Mo1	179.1 (3)	C15—N15—H15A	110.5
N6—C6—Mo1	175.8 (3)	Co2—N15—H15A	110.5
N7—C7—Mo1	178.7 (3)	C15—N15—H15B	110.5
N8—C8—Mo1	175.3 (3)	Co2—N15—H15B	110.5
N13—Co1—N11	90.15 (13)	H15A—N15—H15B	108.7
N13—Co1—N10	93.03 (12)	C16—C15—N15	109.8 (10)
N11—Co1—N10	91.88 (12)	C16—C15—H15C	109.7
N13—Co1—N14	84.34 (12)	N15—C15—H15C	109.7
N11—Co1—N14	174.03 (13)	C16—C15—H15D	109.7
N10—Co1—N14	90.71 (13)	N15—C15—H15D	109.7
N13—Co1—N12	92.07 (12)	H15C—C15—H15D	108.2
N11—Co1—N12	85.60 (12)	C15—C16—N16	108.0 (9)
N10—Co1—N12	174.32 (13)	C15—C16—H16A	110.1
N14—Co1—N12	92.28 (12)	N16—C16—H16A	110.1
N13—Co1—N9	176.73 (12)	C15—C16—H16B	110.1
N11—Co1—N9	92.57 (13)	N16—C16—H16B	110.1
N10—Co1—N9	85.07 (12)	H16A—C16—H16B	108.4
N14—Co1—N9	93.01 (12)	C16—N16—Co2	106.4 (12)
N12—Co1—N9	89.96 (12)	C16—N16—H16C	110.4
C9—N9—Co1	109.2 (2)	Co2—N16—H16C	110.4
C9—N9—H9C	109.8	C16—N16—H16D	110.4
Co1—N9—H9C	109.8	Co2—N16—H16D	110.4
C9—N9—H9D	109.8	H16C—N16—H16D	108.6
Co1—N9—H9D	109.8	C17—N17—Co2	99.6 (12)
H9C—N9—H9D	108.3	C17—N17—H17A	106.5
C10—C9—N9	107.8 (3)	Co2—N17—H17A	105.3
C10—C9—H9A	110.1	C17—N17—H17B	118.8
N9—C9—H9A	109.5	Co2—N17—H17B	111.0
C10—C9—H9B	110.2	H17A—N17—H17B	113.9
N9—C9—H9B	110.7	C18—C17—N17	108.5 (10)
H9A—C9—H9B	108.6	C18—C17—H17C	110.0
C9—C10—N10	108.1 (3)	N17—C17—H17C	110.0
C9—C10—H10A	110.1	C18—C17—H17D	110.0
N10—C10—H10A	110.1	N17—C17—H17D	110.0
C9—C10—H10B	110.1	H17C—C17—H17D	108.4
N10—C10—H10B	110.1	C17—C18—N18	109.5 (11)
H10A—C10—H10B	108.4	C17—C18—H18A	109.8
C10—N10—Co1	108.2 (2)	N18—C18—H18A	109.8
C10—N10—H10C	110.1	C17—C18—H18B	109.8
Co1—N10—H10C	110.1	N18—C18—H18B	109.8
C10—N10—H10D	110.1	H18A—C18—H18B	108.2
Co1—N10—H10D	110.1	C18—N18—Co2	98.7 (12)
H10C—N10—H10D	108.4	C18—N18—H18C	112.0
C11—N11—Co1	111.1 (2)	Co2—N18—H18C	112.0
C11—N11—H11C	109.4	C18—N18—H18D	112.0
Co1—N11—H11C	109.4	Co2—N18—H18D	112.0
C11—N11—H11D	109.4	H18C—N18—H18D	109.7
Co1—N11—H11D	109.4	C19—N19—Co2	97.0 (10)
H11C—N11—H11D	108.0	C19—N19—H19A	112.4
N11—C11—C12	103.9 (3)	Co2—N19—H19A	112.4
N11—C11—H11A	111.0	C19—N19—H19B	112.4
C12—C11—H11A	111.0	Co2—N19—H19B	112.4
N11—C11—H11B	111.0	H19A—N19—H19B	109.9
C12—C11—H11B	111.0	C20—C19—N19	116.9 (10)
H11A—C11—H11B	109.0	C20—C19—H19C	108.1
N12—C12—C11	108.8 (3)	N19—C19—H19C	108.1
N12—C12—H12A	109.9	C20—C19—H19D	108.1
C11—C12—H12A	109.9	N19—C19—H19D	108.1
N12—C12—H12B	109.9	H19C—C19—H19D	107.3
C11—C12—H12B	109.9	C19—C20—N20	104.3 (12)
H12A—C12—H12B	108.3	C19—C20—H20A	110.9
C12—N12—Co1	108.7 (2)	N20—C20—H20A	110.9
C12—N12—H12C	109.9	C19—C20—H20B	110.9
Co1—N12—H12C	109.9	N20—C20—H20B	110.9
C12—N12—H12D	109.9	H20A—C20—H20B	108.9
Co1—N12—H12D	109.9	C20—N20—Co2	103.6 (13)
H12C—N12—H12D	108.3	C20—N20—H20C	104.4
C13—N13—Co1	111.2 (2)	Co2—N20—H20C	112.5
C13—N13—H13C	109.4	C20—N20—H20D	100.3
Co1—N13—H13C	109.4	Co2—N20—H20D	117.6
C13—N13—H13D	109.4	H20C—N20—H20D	115.6
Co1—N13—H13D	109.4	H1A—O1—H1B	109.5
H13C—N13—H13D	108.0	H2A—O2—H2B	109.5
N13—C13—C14	106.6 (3)	H3D—O3—H3C	109.5
N13—C13—H13A	110.4		

Symmetry codes: (i) −*x*+2, −*y*+1, −*z*+1; (ii) −*x*+2, −*y*+1, −*z*.

### Hydrogen-bond geometry (Å, °)

**Table:**

*D*—H···*A*	*D*—H	H···*A*	*D*···*A*	*D*—H···*A*
N9—H9C···N7^iii^	0.92	2.24	3.064 (4)	148
N11—H11C···N7^iii^	0.92	2.09	2.985 (4)	165
N12—H12C···N6^iv^	0.92	2.10	2.955 (4)	154
N13—H13C···N8^iv^	0.92	2.55	3.138 (4)	122
N9—H9D···N2	0.92	2.32	3.117 (4)	145
N14—H14D···N1	0.92	2.28	3.130 (5)	154
N10—H10C···O3^v^	0.92	1.96	2.856 (10)	164
N10—H10D···N5^v^	0.92	2.49	3.125 (5)	126
N11—H11D···N6^v^	0.92	2.58	3.163 (4)	122
N13—H13D···N5^v^	0.92	2.11	3.012 (4)	167
N16—H16C···N4^vi^	0.92	2.52	3.19 (2)	130
N17—H17A···N2^vii^	0.97	2.49	3.29 (2)	140
N20—H20C···N2^vi^	0.85	2.44	3.02 (2)	127
O2—H2A···N8^viii^	0.85 (14)	2.41 (14)	3.207 (9)	156 (12)
O2—H2B···N2^viii^	0.85 (15)	2.50 (15)	3.245 (9)	147 (12)

Symmetry codes: (iii) *x*−1/2, −*y*+1/2, *z*−1/2; (iv) *x*+1/2, −*y*+1/2, *z*−1/2; (v) *x*, *y*, *z*−1; (vi) −*x*+1, −*y*+1, −*z*+1; (vii) *x*+1, *y*, *z*; (viii) −*x*, −*y*+1, −*z*+1.
